# Evaluación del diseño y costo-efectividad de un modelo de pruebas en el lugar de atención para mejorar la capacidad de resolución de las urgencias hospitalarias

**DOI:** 10.1515/almed-2025-0171

**Published:** 2025-11-14

**Authors:** Marta Jimenez-Barragan, Antonio Leon-Justel, Catalina Sanchez-Mora

**Affiliations:** Servicio de Análisis Clínicos, Hospital Universitario Virgen Macarena, Sevilla, España; Instituto de Biomedicina de Sevilla (IBIS), Consejo Superior de Investigaciones Científicas (CSIC), Universidad de Loyola Andalucía - Campus de Sevilla, Universidad Loyola (Andalucía), Sevilla, España

**Keywords:** coste-efectividad, servicio de urgencias, masificación, *point-of-care testing*, tiempo de respuesta

## Abstract

**Objetivos:**

La masificación de los servicios de urgencias, afecta a la calidad asistencial, representando además un problema de carácter económico. Entre sus causas se encuentra el prolongado tiempo de permanencia (*emergency department length of stay*; ED LoS) en el Servicio de Urgencias (SU). Una de las causas identificadas son los prolongados tiempos de respuesta (TR) de las pruebas complementarias, entre las que se incluyen las pruebas analíticas. El objetivo principal de este estudio es diseñar y validar un modelo coste-efectivo que permita mejorar la capacidad de resolución de las urgencias hospitalarias del Hospital Universitario Virgen Macarena (HUVM) basado en la aplicación del *point-of-care testing* (POCT) en pacientes clasificados como de prioridad 3 (P3), de acuerdo con el sistema de triaje del HUVM.

**Métodos:**

Se asignó aleatoriamente a los pacientes P3 que cumplieran los criterios de inclusión en dos grupos: grupo POCT (pruebas analíticas en Urgencias realizadas con dispositivos POCT) o grupo LAB (pruebas analíticas realizadas en el laboratorio central). Anteriormente, se llevó a cabo un estudio de correlación entre parámetros analíticos en ambos grupos. Se analizaron el sexo, la edad, el motivo de la consulta, el tiempo de respuesta (TR) previo a la intervención, el tiempo de decisión sobre la disposición (TDD y ED LoS con o sin pruebas de imagen. Así mismo, se llevó a cabo un estudio de costes y una extrapolación de la estrategia a nivel nacional.

**Resultados:**

El estudio de correlación arrojó resultados favorables. Con el empleo de POCT, se logró una mediana de reducción del TDD y de 107,00 y 118,50 minutos, respectivamente. Esta tendencia se mantuvo en las consultas no relacionadas con el dolor, e independientemente de la realización o no de estudios de imagen. El uso de las POCT resultó en un ahorro de 119,85€/episodio y una razón de coste-efectividad incremental (RCEI) favorable de 60,68 € ahorrados/hora por ED LoS. Según nuestras estimaciones, la implementación de POCT a nivel nacional en el 50 % de las urgencias P3 supondría un ahorro potencial de 284.206.701,19 €.

**Conclusiones:**

Nuestra estrategia demuestra que el empleo de dispositivos POCT redunda en una reducción de TDD y, por consiguiente, del ED LoS, de manera coste-efectiva.

## Introducción

La masificación de los Servicios de Urgencias representa un problema sanitario a nivel global [1], 2], cuyas consecuencias en términos de morbilidad [3], mortalidad [4] y calidad asistencial en los Servicios de Urgencias Hospitalarias (SUH) ha sido objeto de análisis en todo el mundo, habiéndose evidenciado la necesidad de minimizar urgentemente dicho fenómeno [5].

Este problema no compromete únicamente la eficacia operativa de los SUH, sino que también puede llevar al colapso del hospital en su conjunto [6], 7], con consecuencias tanto para los pacientes como para el personal sanitario, conllevando además un incremento de los costes sanitarios.

La masificación de las urgencias constituye un problema económico de primer orden, dado que esta situación es un desajuste entre la oferta y la demanda que resulta insostenible para los sistemas sanitarios. Desde hace tres décadas, estudios como el llevado a cabo por Krochmal and Riley [8], donde se evaluó el incremento del tiempo de permanencia (*emergency department length of stay*; ED LoS) en los SU como medida indirecta, han revelado los enormes costes hospitalarios (6,8 millones de $ en tres años) que este problema supone. Otros estudios, como el de Bayley y col. [9] han demostrado una posible pérdida de ingresos económicos debido a la masificación de los SUH, y un nutrido cuerpo de estudios revela que el crecimiento de la población no sería la única causa de la masificación de las Urgencias [10], [11], [12].

Algunos factores que también influyen en este fenómeno son los retrasos provocados a la espera de recibir los resultados de los estudios complementarios (como las pruebas analíticas), lo que contribuiría en gran medida a prolongar ED LoS [13], provocando la aglomeración de pacientes en dicho Servicio. De hecho, el porcentaje de pacientes atendidos en los SUH que precisan una o más pruebas analíticas es muy elevado, alcanzando alrededor del 75 %.

Los pacientes clasificados como de prioridad 3 (P3) según el sistema de triaje del Hospital Universitario Virgen Macarena (HUVM) *Emergency Severity Index*; ESI, representan el grupo más numeroso de pacientes atendidos en el SUH (aproximadamente el 60 %, con 96.885 episodios de urgencias atendidos).

Los pacientes clasificados como P3 son aquellos que presentan algún signo de gravedad, lo que implica que, pese a existir una elevada probabilidad de que reciban el alta domiciliaria, la mayoría tendrán que someterse a pruebas analíticas que den una orientación sobre su manejo (alta o ingreso). De este modo, su tiempo de espera en el SUH se ve muy condicionado por el tiempo de espera que suponga la realización y comunicación de los resultados de dichas pruebas analíticas, lo que implica una segunda consulta con el médico destinada a cerrar el episodio de manera definitiva, provocando, como consecuencia, una acumulación creciente de pacientes en la sala de espera del SUH.

Tras haber realizado una evaluación clínica, económica y organizativa [14], una de las estrategias propuestas para descongestionar las Urgencias Hospitalarias es la incorporación de dispositivos analíticos en el lugar de atención, *point-of-care testing* (POCT) en el SUH. Las pruebas POCT se definen como aquellas realizadas en el lugar de atención al paciente o cerca del mismo, fuera del laboratorio, y realizadas manualmente, semiautomáticamente o automáticamente por personal ajeno al laboratorio. Los dispositivos POCT han experimentado una evolución sustancial en los últimos años, habiendo mejorado su practicabilidad, y proporcionando actualmente determinaciones analíticas de elevada calidad y, por lo tanto, fiables, con resultados completamente intercambiables con aquellos que se obtendrían con los analizadores de referencia del laboratorio central. Todas estas características permiten que personal ajeno al laboratorio debidamente formado pueda emplear de manera rutinaria los dispositivos POCT, siempre sometidos al control y supervisión de personal especializado del Laboratorio Clínico.

Los objetivos principales de este estudio fueron:

– Diseñar y validar un modelo basado en la tecnología POCT que mejore la capacidad de resolución de las urgencias en los SUH.
– Evaluar el impacto de la tecnología POCT aplicada a una cohorte de pacientes de prioridad P3 atendidos en el SUH del HUVM sobre los tiempos de respuesta (TR) y ED LoS.
– Verificar la utilidad de esta tecnología como estrategia para prevenir y reducir la masificación en el SUH.

Como objetivos secundarios se propusieron los siguientes:

– Verificar la calidad analítica e intercambiabilidad de los resultados obtenidos con los dispositivos POCT empleados en este estudio con aquellos obtenidos con los analizadores de referencia empleados en el Laboratorio de Bioquímica Clínica del HUVM (en adelante, el laboratorio central).
– Análisis de tiempos: entre la admisión y la primera consulta (TR A); entre la primera consulta y la decisión médica [tiempo de decisión sobre la disposición (TDD) y ED LoS asociado al empleo de dispositivos POCT, frente al uso de dispositivos del laboratorio central, en nuestra cohorte de pacientes.
– Evaluar el impacto de la realización de radiografías, ecografías y/o pruebas de tomografía computarizada (TC) tiene en los TR considerados, así como en ED LoS, en nuestra cohorte de pacientes.
– Evaluar el impacto económico que supondría la implementación de esta estrategia en el SUH del HUVM y extrapolar dicho impacto económico al ámbito nacional.

## Materiales y métodos

El estudio se dividió en dos fases principales:

– Análisis de los TR y ED LoS al emplear dispositivos POCT frente al circuito convencional. Llevamos a cabo un estudio prospectivo aleatorizado por conglomerados.
– Análisis económico y extrapolación al ámbito nacional. Se calcularon los siguientes parámetros: costes directos, indirectos (personal de Urgencias y costes de los suministros médicos) y costes globales de la estrategia POCT, y razón de coste-efectividad incremental (RCEI). La RCEI es una herramienta matemática que se emplea en economía de la salud para analizar el coste-efectividad de una intervención en el ámbito de la salud frente a otra alternativa.

**Figura 1:**
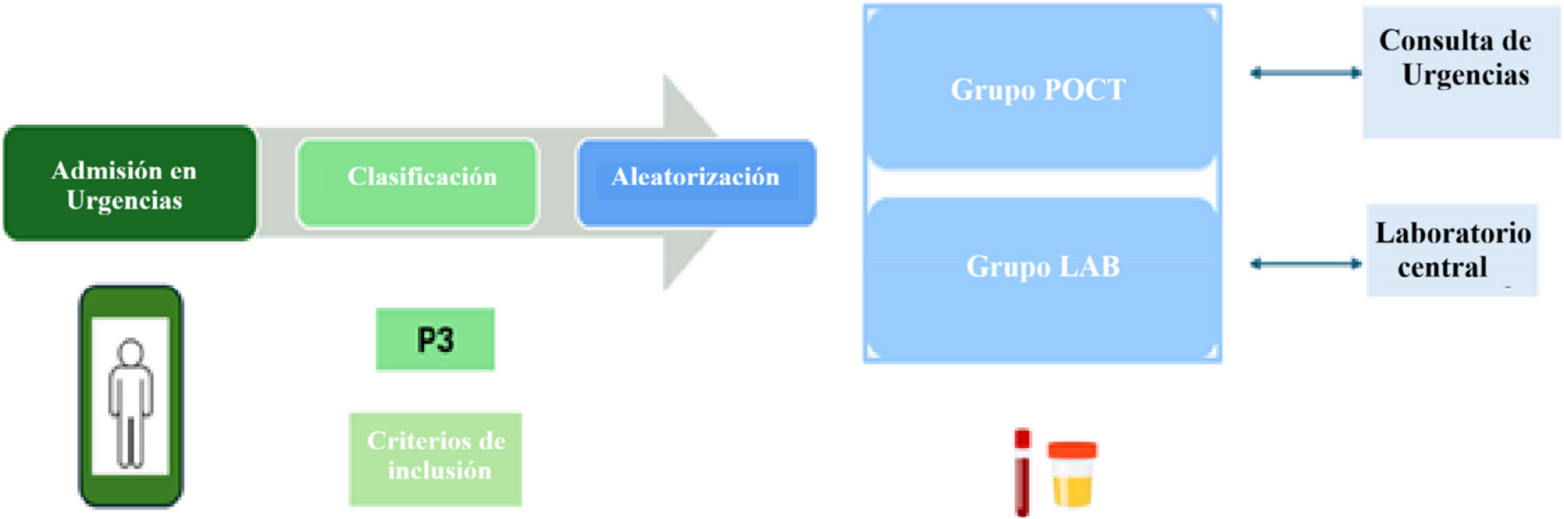
Circuito de pacientes atendidos en el SUH del HUVM y asignación aleatorizada a los grupos de estudio. POCT, *point-of-care testing*; LAB, laboratorio; SUH, Servicio de Urgencias Hospitalarias; P3, prioridad 3; HUVM, Hospital Universitario Virgen Macarena.

El estudio se realizó en el HUVM entre julio de 2023 y junio de 2024, con un periodo de recogida de datos de tres meses, que se extendió de julio a septiembre de 2024. El circuito de los pacientes en el SUH es el siguiente (Figura 1): tras pasar por el mostrador de admisión del SUH, los usuarios clasificados como P3 que cumplieran los criterios de inclusión del estudio fueron asignados aleatoriamente, en la consulta de Urgencias donde fueron atendidos y donde se dispusieron los dispositivos POCT, a uno de los dos grupos de estudio:

– Grupo de intervención (grupo POCT): las pruebas analíticas se realizaron con los dispositivos POCT en la consulta de Urgencias donde se atendió al paciente.
– Grupo de control (grupo LAB): las pruebas analíticas se realizaron en el laboratorio central.

Dependiendo de la sospecha clínica, el médico solicitó las pruebas analíticas pertinentes, en función del perfil acordado entre el SUH y el Laboratorio de Bioquímica Clínica del hospital para este estudio:

– Panel metabólico básico (PMB): glucosa, lactato, creatinina, urea, sodio, potasio y cálculo automático de la tasa de filtración glomerular.
– Hemograma.
– Estudio de coagulación: tiempo de tromboplastina parcial activada (TTPa) y su índice, tiempo de protrombina (TP) y su índice, y la Ratio Internacional Normalizada (*International Normalized Ratio* (INR)) para el grupo LAB/INR para el grupo POCT.
– Estudio del equilibrio ácido-base y de los gases en sangre: gases en sangre venosa.
– Estudio sistemático de orina (incluido el sedimento en el grupo LAB).

En el grupo POCT, se recogieron muestras de sangre total y/u orina, dependiendo del espécimen, en tubos de EDTA dipotásico (K_2_-EDTA), jeringas para gasometría, tubos capilares y/o recipientes para orina. En el grupo LAB, se recogieron muestras de sangre total y/u orina, dependiendo del tipo de espécimen, en tubos con K_2_-EDTA, heparina de litio y/o citrato sódico, jeringas para gasometría y/o recipientes y tubos para orina.

En ambos grupos, los resultados de las pruebas se incorporaron inmediatamente a la historia clínica de los pacientes y el facultativo decidió, según la práctica habitual, el manejo de cada paciente, ya fuera el alta domiciliaria, el ingreso en la sala de observación, o el ingreso en una unidad de hospitalización.

Los sujetos del estudio fueron todos aquellos usuarios adultos que acudieron al SUH del HUVM, fueron clasificados como P3 y que precisaron la realización de una o más de las pruebas analíticas descritas, siendo atendidos en una de las consultas de urgencias de Medicina Interna.

Así, los criterios de inclusión fueron pacientes adultos (≥18 años) clasificados como P3 en el SUH que precisaron las pruebas del laboratorio incluidas en el perfil analítico del estudio y que acudieron a consulta por alguno de los siguientes motivos (entendidos como patologías, signos y/o síntomas):

– Patología respiratoria: descompensación de enfermedad pulmonar obstructiva crónica, síntomas gripales/catarrales, amigdalitis, faringitis, odinofagia, laringitis, sinusitis, ataque de asma, bronquitis, sospecha de neumonía, etc.
– Patología genitourinaria, excluyendo las patologías del aparato reproductor masculino: disuria, uretritis, infección del tracto urinario, sospecha de enfermedad de transmisión sexual (en mujeres), exacerbación de enfermedad renal crónica, sospecha de cólico renal o pielonefritis, dolor en la fosa renal, oliguria, etc.
– Patología abdominal. excluyendo las patologías del hemiabdomen superior o biliopancreáticas: gastroenteritis, reflujo gastroesofágico, vómitos, sospecha de apendicitis, estreñimiento, complicaciones de la enfermedad inflamatoria intestinal, etc.
– Patología hemorrágica: gingivorragia, hematemesis, hemorragia rectal, epistaxis, etc.
– Dolor musculoesquelético: dolor lumbar/cervical no traumático y moderado que no requiere analgesia inmediata, dolor oncológico, dolor inguinal o dolor articular.
– Patología del sistema reproductor masculino: orquitis, epididimitis, bulto, dolor testicular o escrotal, sospecha de enfermedad de transmisión sexual, prostatitis, etc.
– Dolor de cabeza/migraña.
– Patología del sistema cardiovascular, excluyendo cefalea/migraña: crisis hipertensiva, mareos, vértigo, edema, sospecha de síncope vasovagal, descompensación glucémica o parestesia.
– Miscelánea: astenia, fiebre de origen desconocido, confusión, celulitis localizada, trastorno hidroelectrolítico, adenopatía, ansiedad, reacción alérgica, anemia o anorexia/hiporexia.

Se tuvieron en cuenta todos estos motivos de consulta en los pacientes P3, ya que implicaban la realización de pruebas con el perfil analítico descrito y presentaban una elevada probabilidad de recibir el alta domiciliaria.

Los criterios de exclusión, debido a la mayor dificultad manejo en el SUH, fueron: usuarios menores de 18 años, mujeres embarazadas y/o pacientes con trastornos psiquiátricos.

Los criterios de retirada del estudio fueron: reclasificación del paciente a otro nivel de prioridad, asistencia en el circuito de urgencias de traumatología, ginecología u oftalmología y/o necesidad de someterse a pruebas analíticas no contempladas en el estudio o la omisión de petición de pruebas analíticas.

### Métodos analíticos

Los dispositivos POCT empleados y sus métodos analíticos para las diferentes determinaciones fueron los siguientes:

– Analizador ABL90 FLEX PLUS (Radiometer; Brønshøj, Dinamarca): método enzimático amperométrico (glucosa, lactato y creatinina), potenciometría (urea, sodio, potasio, pH y pCO_2_), método óptico/fosforescencia (pO_2_) y espectrofotometría (otros parámetros de oximetría).
– Analizador hematológico pocH-100i (Sysmex; Norderstedt, Alemania): enfoque hidrodinámico y citometría de flujo para la medición de parámetros hematológicos.
– Analizador Urisys 1100 (Roche; Mannheim, Alemania): método de reflectancia fotométrica y refractometría para el análisis de orina.
– Coagulómetro CoaguChek^®^ XS Pro (Roche; Mannheim, Alemania): método coagulométrico para la medición electroquímica del TP tras la activación de la coagulación.

Los analizadores de referencia empleados y sus métodos analíticos para las diferentes determinaciones fueron los siguientes:

– Plataforma analítica modular COBAS 8000 (Roche Diagnostics; Mannheim, Alemania): método enzimático y fotométrico (glucosa), cinética colorimétrica con el método de Jaffé (creatinina), método cinético y fotométrico (urea) y electrodo selectivo de iones (sodio y potasio).
– Analizador ABL 800 FLEX (Radiometer; Brønshøj, Dinamarca): método enzimático amperométrico (lactato), potenciometría (pH y pCO_2_), método óptico/fosforescencia (pO_2_) y espectrofotometría (otros parámetros de oximetría).
– Analizador hematológico XN-1000 (Sysmex; Norderstedt, Alemania): citometría de flujo con impedancia y fluorescencia enfocada hidrodinámicamente para la medición de parámetros hematimétricos.
– Analizador Aution MaxTM AX-4030 (CA. Menarini Diagnostics; Florencia, Italia): método de reflectancia fotométrica para análisis de orina.
– Analizador Sedimax (CA.Menarini Diagnostics; Florencia, Italia): método de refractometría para análisis de orina.
– Analizador de coagulación CS-5100 (Sysmex; Norderstedt, Alemania): método coagulométrico para mediciones electroquímicas de TP y TTPa tras la activación de la coagulación.

### Variables a analizar

– Variables demográficas y clínicas: sexo, edad y motivo de la consulta.
– TR considerando la existencia o no de estudios de imagen, sin tenerlos en cuenta y según el motivo de la consulta: TR A, TDD y ED LoS.
– Costes: personal del SUH (personal médico y de enfermería), suministros médicos, pruebas analíticas, RCEI y potencial ahorro de costes derivados de la implementación progresiva de la estrategia propuesta a nivel nacional.

### Recogida y análisis de datos – métodos estadísticos

La recogida de datos para la primera fase del estudio se llevó a cabo retrospectivamente a través de la historia clínica electrónica de Urgencias y los sistemas informáticos del laboratorio (SIL) y del hospital (SIH). Para el análisis económico, los datos se extrajeron de la literatura científica [15] y del Servicio Andaluz de Salud [16]. Para el análisis del impacto presupuestario estimado a nivel nacional, se recogieron datos extraídos del Sistema de Información de Atención Especializada (SIAE) del Ministerio de Salud, Servicios Sociales e Igualdad (MSSSI).

Con respecto a la evaluación de los dispositivos POCT, se emplearon los siguientes métodos estadísticos: regresión de Passing-Bablok, coeficiente de correlación de Spearman o Pearson y coeficiente de correlación intraclase. La distribución de los datos se examinó mediante la prueba de Shapiro-Wilk. Las variables cuantitativas continuas se expresaron como mediana y rango intercuartílico o como media y desviación estándar, mientras que las variables categóricas se presentaron como recuentos y frecuencias en la población de estudio. Para analizar las diferencias entre las variables cuantitativas continuas independientes, se aplicaron las siguientes pruebas, según el caso: prueba t de Student, prueba *U* de Mann-Whitney o prueba de Kruskal-Wallis. Para las variables categóricas, se empleó la prueba de Chi cuadrado. El nivel de significación <5 % (p<0,05) se consideró estadísticamente significativo.

El tamaño muestral debía permitir la detección de diferencias entre los dos grupos con una potencia del 80 % y un nivel de significación del 5 % utilizando la prueba *t* bilateral y asumiendo una tasa de pérdida durante el seguimiento del 25 %. Así, el tamaño muestral resultante en este estudio fue de 600 episodios de consulta en total, teniendo en cuenta la inclusión de 150 debido a la pérdida prevista del 25 %.

## Resultados y discusión

### Estudio de correlación entre POCT y el laboratorio central

Se llevó a cabo un estudio de correlación en el que se incluyeron los parámetros de interés en un total de 100 muestras por grupo de estudio (POCT y LAB) empleando muestras de sangre pareadas de los pacientes. Dicho estudio no se realizó en los urianálisis, dado que se trata de un método semicuantitativo.

Para llevar a cabo este estudio, se evaluaron los coeficientes de correlación de Spearman y Pearson (dependiendo de la distribución de los datos) y los coeficientes de correlación intraclase, y se realizó un estudio de regresión de Passing-Bablok (Tabla 1).

**Tabla 1:** Estudio comparativo de los resultados obtenidos en los grupos POCT y laboratorio central para los parámetros indicados.

Prueba	Coeficiente de correlación de Spearman o Pearson (IC95 %)	Passing-Bablok		Coeficiente de correlación intraclase (R) (IC95 %)
		Pendiente (IC95 %)	Intersección (IC95 %)	
Creatinina, mg/dL	0,996 (0,985–0,998)	0,9560 (0,8694–1,0085)	0,017 [(−0,031)–0,093]	0,996 (0,992–0,998)
Urea, mg/dL	0,992 (0,979–0,997)	0,9331 (0,9013–1,0074)	0,399 [(−0,356)–1,032]	0,997 (0,984–0,999)
Sodio, mEq/L	0,901 (0,885–0,913)	1,0006 (0,9982–1,2101)	1,003 [(−1,742)–1,019]	0,945 (0,911–0,966)
Potasio, mEq/L	0,992 (0,974–0,995)	1,0001 (0,9078–1,0122)	−0,0840 [(−0,1223)–0,1450]	0,982 (0,874–0,993)
Glucosa, mg/dL	0,996 (0,983–0,999)	0,9643 (0,9556–1,0002)	1,5714 [(−1,8469)–2,9611]	0,991 (0,887–0,994)
Hemoglobina, g/dL	0,992 (0,984–0,995)	1,0000 (0,9857–1,0111)	0,1866 [(−0,2124)–0,4784]	0,993 (0,992–0,998)
Hematíes, × 10^6^/μL	0,993 (0,976–0,994)	1,0047 (0,9858–1,0544)	−0,1002 [(−0,1015)–0,1251]	0,990 (0,985–0,994)
Hematocrito, %	0,985 (0,981–0,989)	1,0438 (0,9859–1,1132)	−2,4816 [(−3,8479)–0,1336]	0,997 (0,975–0,998)
Volumen corpuscular medio, fL	0,952 (0,887–0,976)	1,0163 (0,9441–1,1527)	−2,7522 [(−7,1523)–4,5952]	0,977 (0,956–0,981)
Hemoglobina corpuscular media, pg	0,986 (0,966–0,990)	0,9616 (0,9128–1,1163)	0,9110 [(−0,1421)–1,996]	0,996 (0,992–0,999)
Concentración de hemoglobina corpuscular media, g/dL	0,995 (0,816–0,998)	0,4856 (0,4796–1,0074)	17,5428 [(−0,9588)–18,1365)]	0,986 (0,873–0,993)
Leucocitos, × 10^3^/μL	0,993 (0,982–0,996)	0,9782 (0,9253–1,1310)	0,2132 [(−0,0513)–0,3763]	0,989 (0,979–0,992)
Plaquetas, ×10^3^/μL	0,989 (0,973–0,991)	0,9871 (0,9562–1,0213)	0,6988 [(−10,0866)–19,9285]	0,988 (0,983–0,990)
pH en gasometría (sangre venosa)	0,971 (0,970–0,982)	1,0166 (1,0000–1,1033)	−0,3692 [(−0,6431)–0,0116]	0,994 (0,988–0,997)
Presión parcial de CO_2_ (pCO_2_; venosa)	0,980 (0,962–0,990)	0,9945 (0,9746–1,0152)	3,7815 [(−0,0028)–4,3780]	0,989 (0,986–0,997)
Presión parcial de O_2_ (pO_2_; venosa)	0,946 (0,926–0,963)	1,2619 (0,9985–1,2893)	−3,5547 [(−5,0996)–0,9541]	0,979 (0,896–0,982)
Bicarbonato actual (HCO_3_^−^ actual; venoso)	0,999 (0,981–1,000)	1,0713 (1,0000–1,1116)	−0,8003 [(−1,6869)–0,08416]	0,991 (0,983–0,995)
Bicarbonato estándar (HCO_3_^−^; venoso)	0,986 (0,980–0,993)	1,1258 (0,9986–1,1457)	−2,5221 [(−2,8783)–0,9963]	0,988 (0,981–0,990)
Contenido total de CO_2_ (venoso)	0,982 (0,846–0,995)	1,1156 (0,9714–1,1237)	−0,8014 [(−1,4545)–0,1362]	0,987 (0,979–0,991)
Exceso de bases (en sangre/actual) (venoso)	0,979 (0,974–0,983)	1,1036 (1,0000–1,1269)	0,7490 ([−0,3559)–0,9564)]	0,987 (0,963–0,989)
Exceso de bases (extracelular/estándar) (venoso)	0,985 (0,982–0,999)	1,2327 (0,9693–1,2586)	0,8471 [(−0,2520)–0,9135]	0,995 (0,993–0,998)
Saturación de O_2_ (calculada) (venoso)	0,976 (0,973–0,993)	0,9480 (0,9114–1,0522)	6,7366 [(−0,0117)–8,1749)	0,988 (0,874–0,996)
p50 (venoso)	0,874 (0,772–0,903)	0,9874 (0,8716–1,0243)	1,1813 [(−1,1448)–2,9721]	0,941 (0,933–0,957)
INR	0,987 (0,985–0,999)	0,9744 (0,8956–1,1515)	1,0223 [(−0,0545)–1,0316]	0,992 (0,990–0,997)

IC95 %, intervalo de confianza 95 %. INR, Ratio Internacional Normalizada.

El coeficiente de correlación de Spearman o de Pearson, si aplicaba, fue cercano a 1 para cada prueba (p<0,01). No se observaron diferencias con la regresión de Passing-Bablok entre los dos grupos (IC95 % de la ordenada en el origen contenía el valor 0 y el IC95 % de la pendiente contenía el valor 1). El coeficiente de correlación intraclase también fue cercano a 1.

Por lo tanto, observamos una correlación fuerte y positiva en todos los parámetros evaluados entre los analizadores del laboratorio central y los dispositivos POCT, y las mediciones fueron comparables, ya que, además, su nivel de concordancia fue muy bueno (R>0,90).

### Análisis de TR

En la cohorte del estudio se incluyó a 600 pacientes, 300 por grupo de estudio. El análisis descriptivo mostró homogeneidad en la distribución de sexo, edad y motivos de consulta entre ambos grupos (p>0,050), pero no en la frecuencia de algunas pruebas analíticas y/o estudios de imagen (p<0,050) (Tabla 2).

**Tabla 2:** Características de la cohorte de estudio y análisis univariante.

Características	Total (n=600)	POCT (n=300)	LAB (n=300)	Valor p
Sexo (masculino)	299 (49,83 %)	146 (48,67 %)	153 (51,33 %)	0,624
Edad	56,00 [41,00–67,00]	56,00 [40,75–67,25]	55,00 [41,00–67,00]	0,886

**Motivo de la consulta**				

Patología respiratoria (1)	65 (10,83 %)	32 (10,67 %)	33 (11,00 %)	1,000
Patología genitourinaria (2)	128 (21,33 %)	65 (21,67 %)	63 (21,00 %)	0,921
Patología abdominal (3)	81 (13,50 %)	41 (13,67 %)	40 (13,33 %)	1,000
Patología hemorrágica (4)	72 (12,00 %)	37 (12,33 %)	35 (11,67 %)	0,900
Dolor musculoesquelético (5)	14 (2,33 %)	7 (2,33 %)	7 (2,33 %)	1,000
Patología del aparato genital masculino (6)	24 (4,00 %)	12 (4,00 %)	12 (4,00 %)	1,000
Dolor de cabeza/migraña (7)	29 (4,84 %)	14 (4,66 %)	15 (5,00 %)	1,000
Patología del sistema cardiovascular (8)	69 (11,50 %)	33 (11,00 %)	36 (12,00 %)	0,798
Miscelánea (9)	118 (19,67 %)	59 (19,67 %)	59 (19,67 %)	1,000

**Pruebas analíticas**				

Hemograma	566 (94,33 %)	278 (92,67 %)	288 (96,00 %)	0,112
PMB	551 (91,83 %)	261 (87,00 %)	290 (96,67 %)	<0,050
Gasometría en sangre	119 (19,83 %)	73 (24,33 %)	46 (15,33 %)	<0,050
Urianálisis	219 (36,50 %)	102 (34,00 %)	117 (39,00 %)	0,235
INR	249 (41,50 %)	61 (20,33 %)	188 (62,67 %)	<0,050

**Pruebas de imagen, número de pacientes**	302 (50,33 %)	131 (43,67 %)	171 (56,33 %)	<0,050

RX (número de pruebas)	257 (85,10 %)	109 (83,21 %)	148 (86,55 %)	<0,050
ECO (número de pruebas)	17 (5,63 %)	7 (5,34 %)	10 (5,85 %)	0,623
TC (número de pruebas)	59 (19,54 %)	24 (18,32 %)	35 (20,47 %)	0,170

Los resultados se presentan como mediana y rango intercuartílico para las variables cuantitativas continuas y como recuento y frecuencia (porcentaje, %) para las variables categóricas. POCT, *point-of-care testing*; LAB, laboratorio; INR, *International Normalized Ratio*; RX, radiografía; ECO, ecografía; TC, tomografía computarizada; PMB, panel metabólico básico.

Tal como muestra la Tabla 2, el empleo de sistemas POCT redujo sustancialmente la demanda de estudios de coagulación, aunque el volumen de gasometrías aumentó debido a la presencia de analizadores de gases en sangre en la consulta del SUH. Así mismo, se realizaron menos estudios de imagen en el grupo POCT globalmente, cuya causa requeriría un análisis en profundidad.

En general, observamos diferencias estadísticamente significativas entre los dos grupos en relación a TDD y ED LoS (p<0,050) (Tabla 3), con una mediana de ahorro de 107,00 y 118,50 minutos en el TDD y ED LoS, respectivamente, en el grupo POCT. Por otro lado, no hubo diferencias significativas en el TR A, lo cual es lógico, dado que corresponde a un momento temporal anterior a la intervención POCT propuesta, por lo que no se vio afectado por la misma.

**Tabla 3:** Análisis de TR A, TDD y ED LoS en toda la cohorte global.

Cohorte global											
TR A, minutos			Valor p	TDD, minutos			Valor p	ED LoS, minutos			Valor p
Total	POCT	LAB		Total	POCT	LAB		Total	POCT	LAB	
40,75 [22,50–69,00]	40,00 [21,75–70,00]	41,50 [23,00–65,50]	0,912	176,50 [111,75–259,25]	122,00 [81,50–196,00]	229,00 [168,00–390,50]	<0,050	235,00 [158,00–341,00]	179,50 [120,75–252,25]	298,00 [228,50–469,25]	<0,050

En función del motivo de consulta									
	TR A, minutos		Valor p	TDD, minutos		Valor p	ED LoS, minutos		Valor p
	POCT	LAB		POCT	LAB		POCT	LAB	
1	53,00 [30,00–93,00]	57,00 [39,00–89,00]	0,922	115,00 [90,00–192,00]	189,00 [150,00–249,00]	<0,050	186,50 [146,50–268,25]	257,00 [202,00–334,00]	<0,050
2	39,00 [19,00–65,00]	47,00 [36,00–71,00]	0,155	114,00 [79,00–181,00]	199,00 [134,00–253,00]	<0,050	162,00 [117,00–239,00]	250,00 [200,00–320,00]	<0,050
3	31,00 [16,00–56,00]	68,00 [40,00–101,00]	0,147	110,00 [80,00–205,00]	208,00 [150,00–277,00]	<0,050	150,00 [116,00–252,00]	277,00 [216,75–359,50]	<0,050
4	38,00 [25,00–56,00]	47,00 [28,00–69,00]	0,132	92,00 [66,00–168,00]	212,00 [174,00–293,00]	<0,050	143,00 [96,00–210,00]	276,00 [203,00–377,50]	<0,050
5	47,00 [37,00–68,00]	38,00 [21,00–52,00]	0,339	151,00 [133,00–190,00]	168,00 [154,00–214,00]	0,352	201,00 [186,50–239,50]	206,00 [179,00–264,50]	0,917
6	37,00 [29,00–62,00]	91,00 [33,00–123,00]	0,060	108,00 [96,00–156,00]	201,00 [173,00–235,00]	<0,050	188,00 [123,75–225,25]	277,00 [241,75–352,25]	<0,050
7	53,00 [41,00–77,00]	78,00 [42,00–88,00]	0,085	153,00 [114,00–271,00]	207,00 [200,00–281,00]	0,112	202,50 [161,00–334,50]	283,50 [241,50–310,50]	0,051
8	52,00 [17,00–88,00]	58,00 [27,00–96,00]	0,274	171,00 [106,00–219,00]	227,00 [165,50–303,00]	<0,050	225,00 [146,00–308,00]	296,00 [207,75–364,75]	<0,050
9	49,00 [25,00–71,00]	56,00 [31,00–98,00]	0,117	132,00 [72,00–173,00]	243,00 [211,50–315,00]	<0,050	180,00 [112,00–268,50]	301,00 [245,50–465,25]	<0,050

Los resultados se expresan como mediana y rango intercuartílico. TR, tiempo de respuesta; TDD, tiempo de decisión de disposición; ED LoS, *emergency department length of stay*; POCT, *point-of-care testing*; LAB, laboratorio. La numeración está vinculada a cada uno de los motivos de consulta considerados en el estudio. Los números proceden de la Tabla 2.

Con respecto al motivo de consulta (Tabla 3), observamos la misma tendencia, excepto en los grupos 5 “Dolor musculoesquelético” y 7 “Dolor de cabeza/migraña”, tal como muestra la Tabla 2, en los que no identificamos diferencias significativas en ninguno de los tres periodos.

En estos dos casos, el TDD y ED LoS se ven fuertemente influidos por factores no analíticos, viéndose especialmente condicionados por el tiempo transcurrido hasta lograr un buen control del dolor en el paciente, lo que significa que la tecnología POCT no acorta estos tiempos significativamente. Además, el tamaño de la muestra para estos dos motivos de consulta es limitado, lo que podría afectar también a la evaluación de sus TR y Ed LoS.

La realización o no de estudios de imagen no provocó diferencias significativas entre los grupos de estudio en ninguno de los tres periodos analizados, manteniéndose la tendencia descrita en el análisis de la cohorte total (Tabla 4).

**Tabla 4:** Análisis del TR A, TDD y ED LoS en la cohorte de pacientes que precisaron/no precisaron estudios de imagen.

**Estudios de imagen**				

**TR, minutos**	**Total (n=302)**	**POCT (n**=**131)**	**LAB (n**=**171)**	**Valor p**

TR A	40,75 [24,25–86,00]	39,00 [18,50–66,50]	40,25 [32,00–94,00]	0,863
TDD	202,00 [136,50–287,00]	155,00 [106,50–219,50]	229,00 [180,00–384,50]	<0,050
ED LoS	259,00 [189,00–365,25]	208,00 [141,00–282,50]	302,00 [233,00–453,50]	<0,050

Sin estudios de imagen				
TR, minutos	Total (n=298)	POCT (n=169)	LAB (n=129)	Valor p
TR A	45,50 [27,00–79,00]	42,00 [24,00–72,00]	40,25 [31,00–89,00]	0,763
TDD	153,00 [88,00–235,75]	99,00 [69,00–161,00]	227,00 [161,00–398,00]	<0,050
ED LoS	205,00 [137,25–302,50]	157,00 [99,00–225,00]	296,00 [202,00–477,00]	<0,050

TR, tiempo de respuesta; TDD, tiempo de decisión de disposición; ED LoS, *emergency department length of stay*; POCT, *point-of-care testing*; LAB, laboratorio.

### Análisis económico

Los costes en euros de 2019, tomando como referencia los datos proporcionados por el Servicio Andaluz de Salud [16].

El coste de la atención médica en el SUH se evaluó teniendo en cuenta el personal de Urgencias y los suministros médicos empleados. Los costes de personal de Urgencias se calcularon en función de los salarios establecidos en las tablas salariales del Sistema Andaluz de Salud [16]. Para calcular los costes de los suministros médicos, se emplearon datos procedentes de fuentes internas del hospital, quedando excluidos los gastos de farmacia. En ambos casos, se siguieron las recomendaciones de Schilling [17], también contempladas en el artículo de Goldstein y col. [15]. Según dicho método, el coste por minuto del personal de Urgencias se calculó de la siguiente manera: en primer lugar, calculamos el coste total del personal de Urgencias dividido entre los minutos totales anuales. Se aplicó la presunción de que la dotación de personal presentaría una distribución uniforme a lo largo del año, habiendo sido tenidos en cuenta únicamente los costes asociados al personal médico y de enfermería. De este modo, se calculó que el coste por minuto del personal de Urgencias fue de 28,60 euros.

Posteriormente, siguiendo la recomendación de Schilling, el número de pacientes presentes simultáneamente en nuestro SUH se calculó dividiendo los minutos de un año entre el número de urgencias P3 atendidas anualmente en nuestro hospital, lo que arrojó un tiempo medio de 5,4 minutos entre la llegada de un paciente y el siguiente. De este modo, teniendo en cuenta el ED LoS medio en la práctica estándar (LAB), que es de 298,00 minutos, esto significa que en nuestro SUH había:

298,00/5,4=55 pacientes en el SUH en cualquier momento.

Por lo tanto, los costes de dotación de personal por paciente fueron los siguientes:

(28,60 €/minuto)/55 pacientes=0,52 € por minuto por paciente.

Así mismo, calculamos los costes de los suministros médicos por minuto y por paciente. Teniendo en cuenta que se calculó que el coste de un minuto de suministros médicos en el SUH fue de 1,99 euros, esto equivalía a 0,037 euros por paciente por minuto.

Calculamos la diferencia media para cada coste entre los dos grupos (POCT - LAB) y estos costes fueron también contrastados. Finalmente, comparamos el coste total entre los dos grupos, teniendo en cuenta tanto los costes directos (pruebas analíticas) como los indirectos (personal y suministros médicos del SUH) (Tabla 5).

**Tabla 5:** Análisis de costes del grupo POCT y del grupo LAB.

Costes, €	POCT (n=300)	LAB (n=300)	Costes incrementales	Valor p
Suministros médicos	7,33 (6,87–7,79)	15,60 (14,26–16,94)	−8,27 [−9,73)–( −6,81)]	<0,050
Personal del SUH	103,04 (96,54–109,54)	219,24 (200,35–238,13)	−116,20 [(−136,68)–( −95,73)]	<0,050
Pruebas analíticas	5,81 (5,59–6,02)	1,18 (1,13–1,24)	4,63 (4,40–4,85)	<0,050
Costes totales	116,18 (109,21–123,14)	236,02 (215,79–256,25)	−119,85 [(−141,78)–(−97,91)]	<0,050

Costes expresados en euros/episodio atendido en el SUH (media e IC95 %). POCT, *point-of-care testing*; LAB, laboratorio.

En el análisis económico, se obtuvieron los siguientes costes:

Aunque los costes directos (pruebas analíticas) fueron mayores en el grupo POCT frente al grupo LAB, globalmente, el empleo de POCT resultó en un ahorro de 119,85 euros/episodio, ya que los costes indirectos (suministros médicos y personal del SUH) fueron menores.

El RCEI fue calculado de la siguiente manera:

RCEI: Coste incremental total/mediana incremental ED LoS.

Tal como muestra la Tabla 3, la mediana de ED LoS fue de 179,50 minutos y 298,00 en los grupos POCT y LAB, respectivamente. Por lo tanto, la mediana incremental de ED LoS es de −118,5 minutos.

Por lo tanto:

RCEI: (−119,85) €/(−118,5) min=1,0114 €/minuto.

De este modo, el cálculo para 60 minutos fue:

1,0114 €/minuto × 60 minutos=60,68 € ahorrados/hora ED LoS.

Así, obtuvimos un RCEI favorable de 60,68 € ahorrados/hora ED LoS.

Finalmente, se realizó una extrapolación del impacto económico que tendría a nivel nacional la implementación de la estrategia POCT considerada en este estudio. Utilizando datos del MSSSI (SIAE). El número total de consultas en Urgencias en España fue de 30.372.076, siendo aproximadamente la mitad de ellas clasificadas como urgencias P3 (15 186 043). Con la aplicación de la tecnología POCT en el 50 % de las urgencias P3 a nivel nacional, se estimó un ahorro potencial de 284.206.701,19 euros.

En resumen, con la estrategia presentada para mejorar la capacidad de resolución de los SUH consistente en la aplicación de sistemas POCT en pacientes clasificados como P3 en SUH, hemos verificado una reducción de uno de los TR que más influyen en el tiempo de permanencia global de los pacientes en las Urgencias, el TDD y, en consecuencia, en el ED LoS.

Tal fue el caso de la mayor parte de los motivos de consulta analizados, independientemente de la realización o no de estudios de imagen. Cabe señalar la gran importancia de haber centrado la estrategia en los pacientes P3, dado que estos constituyen la mayoría de los usuarios atendidos en el SUH. Además, la mayoría de estos pacientes tienen gran probabilidad de recibir el alta domiciliaria tras ser atendidos, precisando la mayoría de ellos un panel básico de pruebas analíticas que se pueden realizar sin dificultad con los dispositivos POCT disponibles.

Además, ha demostrado ser una estrategia coste-efectiva, dado que los costes médicos totales de la atención médica en los costes totales de la atención sanitaria en el grupo de intervención fueron inferiores a los del grupo de control.

Si bien es cierto que la robustez de nuestras conclusiones puede verse limitada en cuanto a su extrapolación nacional, dado que se trata de un estudio realizado en un único centro hospitalario y que, por tanto, debería validarse en el centro en cuestión antes de implementar la estrategia propuesta, la extrapolación de la intervención POCT a nivel nacional indica un importante ahorro potencial de gracias al uso exclusivo de sistemas POCT en el 50 % de los pacientes P3 atendidos.

Por lo tanto, podemos concluir que nuestra estrategia podría contribuir potencialmente a prevenir y reducir la aglomeración de los SUH de una manera coste-efectiva.
